# Supplementary material for: Exposure-related, global alterations in innate and adaptive immunity; a consideration for re-use of non-human primates in research
Source: PeerJ. 2021 Mar 8;9:e10955. doi: 10.7717/peerj.10955 (PMC7950202; doi:10.7717/peerj.10955)
Supplement: Table S1 [file peerj-09-10955-s002.docx]

**Supplemental Table S1. Statistics of frequency and cell counts of innate immune cells**

|  | **Naïve**  **(n=9)** | **Days post-last vaccination/boost (n=5)** | | | | |
| --- | --- | --- | --- | --- | --- | --- |
|  |  | **6 days** | **20 days** | **2.5 months** | **4 months** | **6 months** |
| gp96-Ig-PfCA - HLA-DR+CD14+ monocytes | | | | | |  |
| Frequency | 4.6±1.9 | 6.7±2.1 *P=0.08* | 5.5±2.6  *P=0.45* | 5.2±2.8  *P=0.64* | 4.2±2.2  *P=0.73* | 5.4±1.9  *P=0.47* |
| Cell counts | 696±521 | 638±315  *P=0.82* | 540±324  *P=0.55* | 476±332  *P=0.41* | 395±284  *P=0.25* | 557±279  *P=0.59* |
| gp96-Ig-PfCA - HLA-DR+NKT cells | | | | | |  |
| Frequency | 15.9±12.6 | 10.3±9.9  *P=0.41* | 13.5±5.4  *P=0.69* | 4.1±3.1  *P=0.06* | 4.8±3.0  *P=0.08* | 7.2±4.8  *P=0.17* |
| Cell counts | 6.6±5.2 | 1.3±0.7  *P=0.01** | 3.4±2.0  *P=0.22* | 0.8±0.4  *P=0.01** | 1.1±0.5  *P=0.01** | 1.6±1.6  *P=0.02** |
| D/Ad-PfCA - HLA-DR+CD14+ monocytes | | | | | | |
| Frequency | 4.6±1.9 | 6.9±2.5  *P=0.07* | 8.0±1.7  *P=0.006*** | 6.2±2.2  *P=0.17* | 7.7±3.0  *P=0.03** | 6.0±0.8  *P=0.15* |
| Cell counts | 696±521 | 690±625  *P=0.98* | 604±324  *P=0.72* | 473±339  *P=0.35* | 604±234  *P=0.71* | 649±251  *P=0.85* |
| D/Ad-PfCA - HLA-DR+NKT cells | | | | | | |
| Frequency | 15.9±12.6 | 6.6±5.8  *P=0.15* | 8.2±4.1  *P=0.12* | 5.2±2.2  *P=0.03** | 3.2±3.4  *P=0.01** | 1.9±1.3  *P=0.01** |
| Cell counts | 6.6±5.2 | 1.6±1.4  *P=0.02** | 2.5±1.8  *P=0.11* | 1.6±0.5  *P=0.02** | 0.8±0.6  *P=0.01** | 0.6±0.2  *P=0.01** |
| ***** P at 0.05 alpha level, unpaired T-test (with welch’s correction when applicable), two-tailed, data represent mean ± standard deviation. | | | | | | |
