# Supplementary material for: Exposure-related, global alterations in innate and adaptive immunity; a consideration for re-use of non-human primates in research
Source: PeerJ. 2021 Mar 8;9:e10955. doi: 10.7717/peerj.10955 (PMC7950202; doi:10.7717/peerj.10955)
Supplement: Table S2 [file peerj-09-10955-s003.docx]

**Supplemental Table S2. Statistics of frequency and cell counts of B cells**

**DPIV/TDENV-LAV vaccine (n=10)**

|  | **Naïve**  **(n=9)** | **1 month** | **2 month** | **3 months** | **4 months** | **6 months** |
| --- | --- | --- | --- | --- | --- | --- |
| Frequency | 32.2±8.7 | 23.0±5.0  *P=0.01** | 18.8±6.8  *P=0.001*** | 20.8±6.7  *P=0.005*** | 17.4±5.8  *P=0.0004**** | 17.3±4.3  *P=0.0002**** |
| Cell counts | 4438±2626 | NA | NA | NA | 1517±1027  *P=0.004*** | 1900±702  *P=0.009*** |
| **gp96-Ig-PfCA (n=5)** | | | | | | |
|  | **Naïve**  **(n=9)** | **6 days** | **20 days** | **2.5 months** | **4 months** | **6 months** |
| Frequency | 32.2±8.7 | 25.1±10.6  *P=0.19* | 27.7±11.7  *P=0.42* | 26.3±0.5  *P=0.28* | 24.6±7.2  *P=0.11* | 23.8±8.5  *P=0.10* |
| Cell counts | 4438±2626 | 2467±1976  *P=0.17* | 2860±2678  *P=0.3* | 2116±1379  *P=0.09* | 2022±996  *P=0.07* | 2394±1194  *P=0.12* |
| **D/Ad-PfCA (n=5)** | | | | | | |
|  | **Naïve**  **(n=9)** | **6 days** | **20 days** | **2.5 months** | **4 months** | **6 months** |
| Frequency | 32.2±8.7 | 18.8±5.5  *P=0.009*** | 20.5±8.4  *P=0.03** | 24.0±10.1  *P=0.13* | 20.0±9.3  *P=0.03** | 19.9±5.0  *P=0.01** |
| Cell counts | 4438±2626 | 1592±955  *P=0.03** | 1538±965  *P=0.03** | 1913±1627  *P=0.07* | 1584±813  *P=0.01** | 2169±983  *P=0.09* |
| ***** P at 0.05 alpha level, unpaired T-test (with welch’s correction when applicable) two-tailed, data represent mean ± standard deviation. NA-Data not available | | | | | | |
