# Supplementary material for: Exposure-related, global alterations in innate and adaptive immunity; a consideration for re-use of non-human primates in research
Source: PeerJ. 2021 Mar 8;9:e10955. doi: 10.7717/peerj.10955 (PMC7950202; doi:10.7717/peerj.10955)
Supplement: Table S3 [file peerj-09-10955-s004.docx]

**Supplemental Table S3. Statistics of frequency and cell counts of activated T cells in naïve group and gp96-Ig-PfCA vaccinated animals**

|  | **Naïve**  **(n=9)** | **Days post-last gp96-Ig-PfCA boost (n=5)** | | | | |
| --- | --- | --- | --- | --- | --- | --- |
|  |  | **6 days** | **20 days** | **2.5 months** | **4 months** | **6 months** |
| Activated T cells (HLA-DR+CD3+) | | | | | |  |
| Frequency | 3.3±1.2 | 2.0±0.9 *P=0.07* | 1.9±0.5  *P=0.04** | 2.0±0.5  *P=0.05** | 1.4±0.4  *P=0.007*** | 1.5±0.3  *P=0.01** |
| Cell counts | 204±149 | 98±65  *P=0.16* | 91±50  *P=0.13* | 86±44  *P=0.05** | 63±37  *P=0.02** | 71±28  *P=0.02** |
| CD3+ gated HLA-DR+CD4+ T cells | | | | | |  |
| Frequency | 2.1±0.7 | 1.7±0.7  *P=0.30* | 1.6±0.5  *P=0.12* | 1.8±0.4  *P=0.28* | 1.2±0.2  *P=0.01** | 1.3±0.2  *P=0.02** |
| Cell counts | 83±52 | 50±27  *P=0.22* | 45±23  *P=0.15* | 45±21  *P=0.15* | 34±18  *P=0.06* | 36±15  *P=0.03** |
| CD3+ gated HLA-DR+CD8+ T cells | | | | | | |
| Frequency | 5.0±2.3 | 3.2±3.4  *P=0.27* | 2.9±2.0  *P=0.11* | 2.5±1.2  *P=0.04** | 1.7±0.9  *P=0.01** | 2.0±0.8  *P=0.01** |
| Cell counts | 80±62 | 36±35  *P=0.17* | 33±23  *P=0.13* | 28±20  *P=0.04** | 18±12  *P=0.01** | 22±10  *P=0.02** |
| ***** P at 0.05 alpha level, unpaired T-test (with welch’s correction when applicable) two-tailed, data represent mean ± standard deviation. | | | | | | |
