# Supplementary material for: Exposure-related, global alterations in innate and adaptive immunity; a consideration for re-use of non-human primates in research
Source: PeerJ. 2021 Mar 8;9:e10955. doi: 10.7717/peerj.10955 (PMC7950202; doi:10.7717/peerj.10955)
Supplement: Table S4 [file peerj-09-10955-s005.docx]

**Supplemental Table S4. Statistics of frequency and cell counts of activated T cells in naïve group and D/Ad-PfCA vaccinated animals**

|  | **Naïve**  **(n=9)** | **Days post-last Ad5 boost (n=5)** | | | | |
| --- | --- | --- | --- | --- | --- | --- |
|  |  | **6 days** | **20 days** | **2.5 months** | **4 months** | **6 months** |
| Activated T cells (HLA-DR+CD3+) | | | | | |  |
| Frequency | 3.3±1.2 | 7.1±3.3  *P=0.06* | 5.3±0.8  *P=0.01** | 4.7±1.0  *P=0.06* | 3.4±0.9  *P=0.91* | 4.4±1.8  *P=0.21* |
| Cell counts | 204±149 | 316±259  *P=0.31* | 178±97  *P=0.72* | 169±91  *P=0.60* | 141±47  *P=0.31* | 214±92  *P=0.87* |
| CD3+ gated HLA-DR+CD4+ T cells | | | | | |  |
| Frequency | 2.1±0.7 | 4.7±2.3  *P=0.06* | 3.7±1.5  *P=0.02** | 3.8±1.0  *P=0.004*** | 2.9±0.9  *P=0.12* | 4.0±2.4  *P=0.16* |
| Cell counts | 83±52 | 95±56  *P=0.69* | 66±33  *P=0.51* | 80±48  *P=0.92* | 64±30  *P=0.47* | 104±46  *P=0.45* |
| CD3+ gated HLA-DR+CD8+ T cells | | | | | | |
| Frequency | 5.0±2.3 | 9.4±6.3  *P=0.2* | 6.3±0.5  *P=0.15* | 5.0±1.8  *P=0.99* | 3.5±1.7  *P=0.22* | 4.7±2.1  *P=0.81* |
| Cell counts | 80±62 | 141±145  *P=0.4* | 60±51  *P=0.56* | 47±39  *P=0.31* | 34±40  *P=0.16* | 45±45  *P=0.25* |
| ***** P at 0.05 alpha level, unpaired T-test (with welch’s correction when applicable) two-tailed, data represent mean ± standard deviation. | | | | | | |
