# Supplementary figures and images for: Exposure-related, global alterations in innate and adaptive immunity; a consideration for re-use of non-human primates in research
Source: PeerJ. 2021 Mar 8;9:e10955. doi: 10.7717/peerj.10955 (PMC7950202; doi:10.7717/peerj.10955)

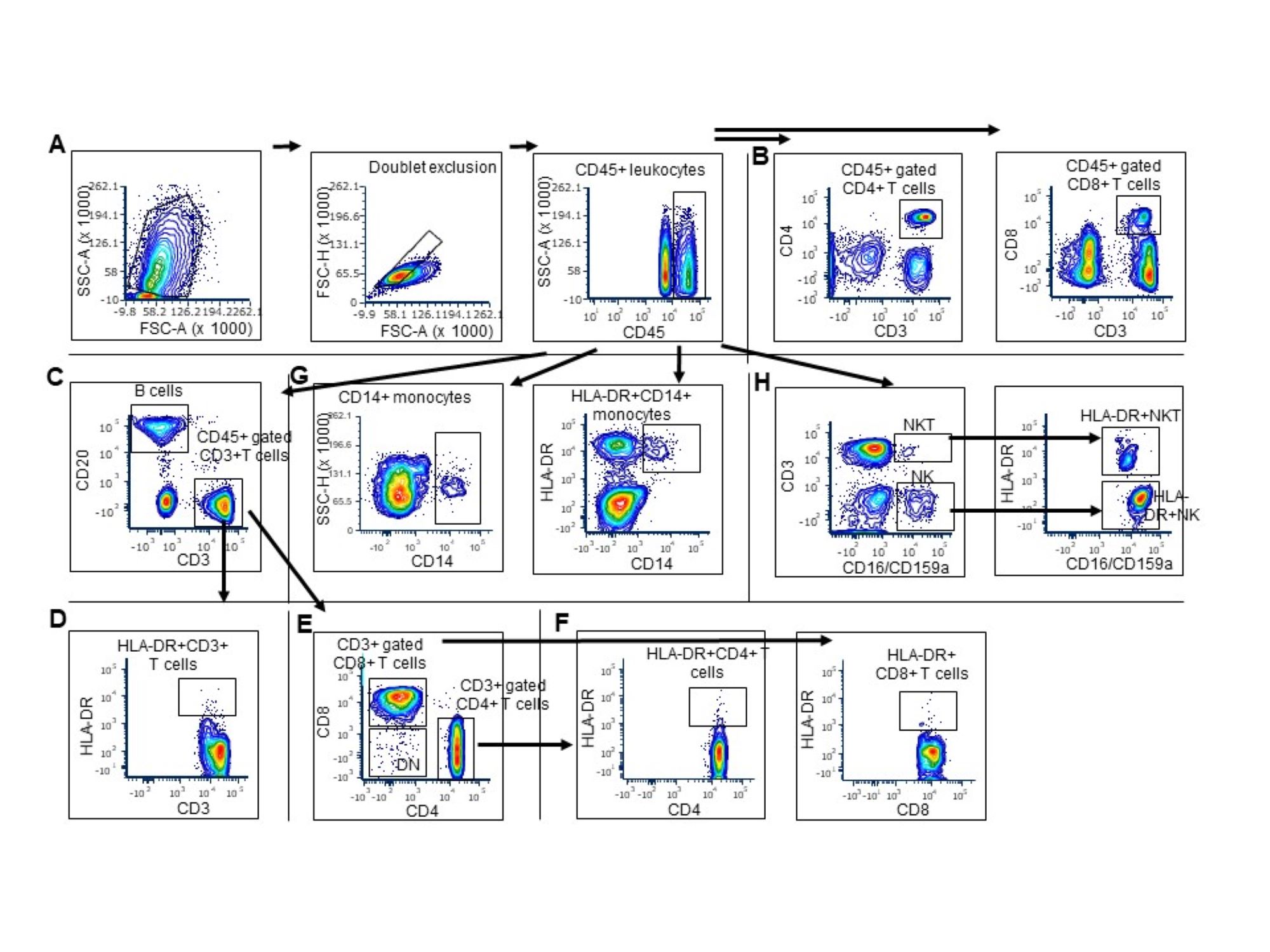

Supplement: Figure S1 — Acquisition was limited to cells expressing V450 fluorochrome/CD45 (common leukocyte antigen) (trigger) at a particle cut-off size (FSC) of 4000. Diluted whole blood samples were acquired at a medium flow rate (50,000 events/sample) by 17-color, LSRII Fortessa flow cytometer at the WRAIR Flow Cytometry core facility. (A) After excluding doublets, leukocytes were gated on side-scatter and CD45. Of the two CD45 populations shown in S1A here, only the CD45hi population expressed all the other lineage markers for innate and adaptive immune cells subsets. Therefore, CD45hi population was selected for further characterization of following cell subsets (B) CD45+ gated CD4+ T cells and CD8+ T cells (C) CD45+ gated CD20+ B cells and CD45+ gated CD3+T cells (D) CD3+ gated activated HLA-DR+CD3+ T cells (E) CD3+ gated CD4+ T cells, CD3+ gated CD8+ T cells, CD4-CD8- double negative (DN) T cells (F) CD3+ gated, activated HLA-DR+CD4+ T cells and HLA-DR+CD8+ T cells (G) CD45+ gated CD14+ monocytes and CD45+ gated HLA-DR+CD14+ monocytes (H) CD45+ gated CD3-CD16/CD159a+ NK cells, CD45+ gated CD3-CD16/CD159a+HLA-DR+ NK cells, CD45+ gated CD3+CD16/CD159a+ NKT cells, CD45+ gated CD3+CD16/CD159a+HLA-DR+ NKT cells. Gating strategy for dendritic cells (DCs) is not shown here. CD45+ leukocytes co-expressing HLA-DR and CD11c were considered as DCs after excluding the cells expressing CD14 and CD16/CD159a, CD3 and CD20 (CD45+CD14-CD16/CD159a-CD3-CD20-CD11c+HLA-DR+). [file peerj-09-10955-s006.jpg]

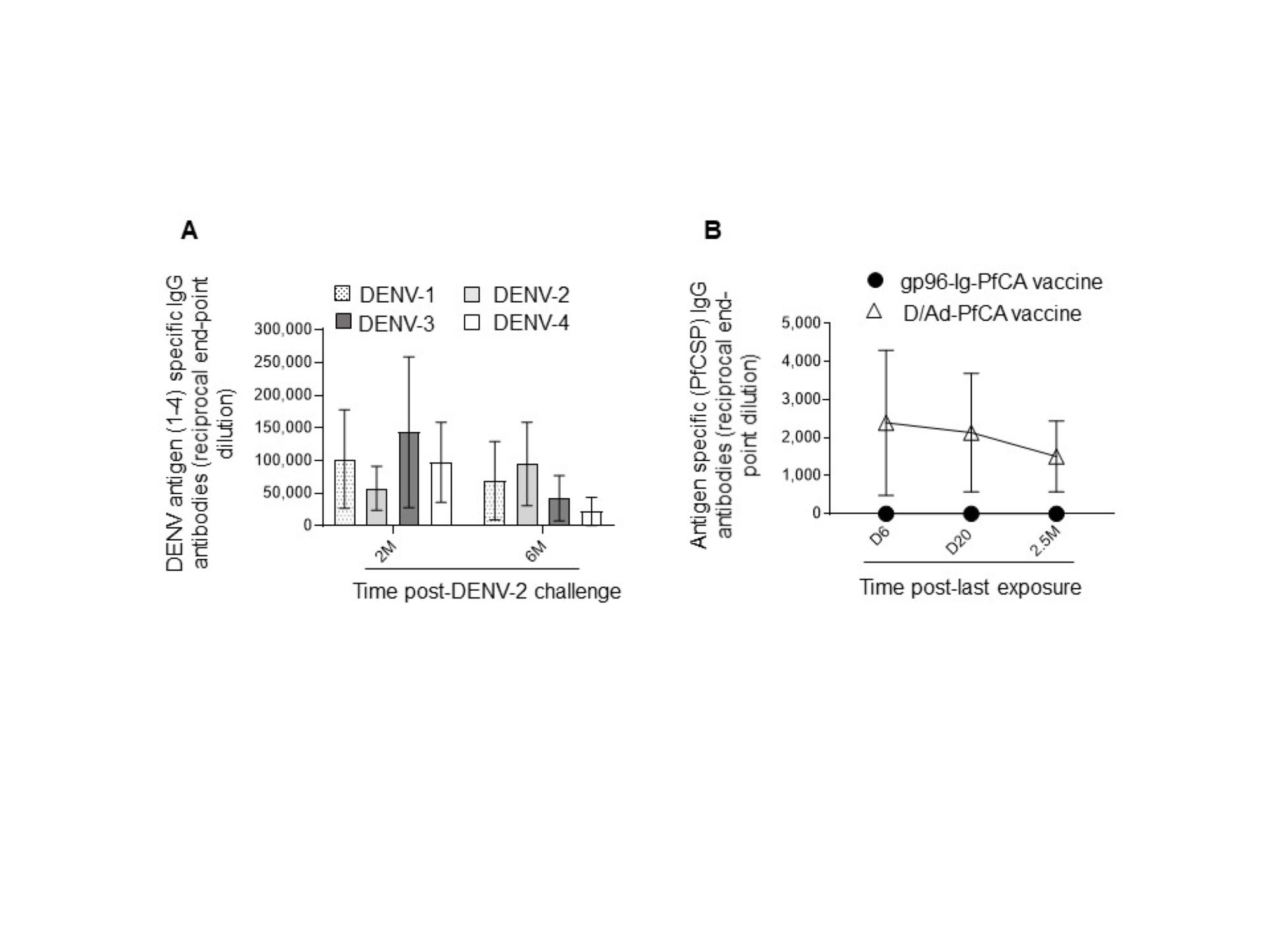

Supplement: Figure S2 — (A) Specific to each of the four dengue serotypes (DENV 1-4) in DPIV/TDENV-LAV vaccinated animals (n=10) at 2 months and 6 months post-DENV-2 challenge (B) specific to Plasmodium falciparum (Pf) full-length circumsporozoite (CSP) protein (PfCSP) in gp96-Ig-PfCA vaccinated animals (n=5) and D/Ad-PfCA vaccinated animals (n=5) at 6 days, 20 days and 2.5 months post-last vaccination. Data represent mean and the standard deviation. [file peerj-09-10955-s007.jpg]
